# Influence of Hydraulic Pressure on Performance Deterioration of Direct Contact Membrane Distillation (DCMD) Process

**DOI:** 10.3390/membranes9030037

**Published:** 2019-03-06

**Authors:** Seung-Min Park, Sangho Lee

**Affiliations:** 1 Environmental Convergence Technology Center, Korea Testing Laboratory 87, Digital-ro 26-gil, Guro-gu, Seoul 08389, Korea; jrpeter@ktl.re.kr; 2 School of Civil and Environmental Engineering, Kookmin University, 861-1, Jeongneung-dong, Seongbuk-gu, Seoul 136-702, Korea

**Keywords:** direct contact membrane distillation (DCMD), hydrophobic membrane, hydraulic pressure, compaction, membrane structure, wetting

## Abstract

Direct contact membrane distillation (DCMD) is a membrane distillation (MD) configuration where feed and distillate directly contact with a hydrophobic membrane. Depending on its operating conditions, the hydraulic pressures of the feed and distillate may be different, leading to adverse effects on the performance of the DCMD process. Nevertheless, little information is available on how hydraulic pressure affects the efficiency of DCMD. Accordingly, this paper investigates the effect of external hydraulic pressure on the process efficiency of DCMD. Gas permeabilities of MD membranes were measured to analyze the effect of membrane compaction by external pressure. Mass transfer coefficients were calculated using experimental data to quantitatively explain the pressure effect. Experiments were also carried out using a laboratory-scale DCMD set-up. After applying the pressure, the cross-sections and surfaces of the membranes were examined using a scanning electron microscope (SEM). Results showed that the membrane structural parameters such as porosity and thickness were changed under relatively high pressure conditions (>30 kPa), leading to reduction in flux. The mass transfer coefficients were also significantly influenced by the hydraulic pressure. Moreover, local wetting of the membranes were observed even below the liquid entry pressure (LEP), which decreased the rejection of salts. These results suggest that the control of hydraulic pressure is important for efficient operation of DCMD process.

## 1. Introduction

Membrane distillation (MD) is a non-isothermal process that uses vapor pressure difference as a driving force for separation of water or volatile chemicals [1,2]. A micro porous hydrophobic membrane prevents the passage of liquids and allows vapor transport. Depending on the arrangement of the distillate channel or the manner in which this channel is operated, there are several different MD configurations, including direct contact MD (DCMD), air gap MD (AGMD), vacuum MD (VMD), sweeping gas MD (SWGMD), vacuum multi-effect membrane distillation (V-MEMD), and permeate gap MD (PGMD). Among them, DCMD has been preferentially studied in bench-scale systems due to its simplicity and straightforwardness.

Recently, MD has attracted increasing attention in both academia and industry. This is because MD has unique advantages over conventional desalination technologies such as reverse osmosis (RO), multi-stage flash (MSF), and multi-effect distillation (MED). One of them is its ability to utilize low-grade thermal energy such as waste heat from power stations and chemical plants. Since the operating temperature of MD is relatively low compared with MSF and MED, it can be operated by low-grade heat sources. Another advantage is its higher rejection against non-volatile solutes such as inorganic ions than RO. This feature enables the production of high-quality water from MD processes. In addition, MD is less sensitive to the concentration of dissolved solutes in feed than RO, allowing MD to be used for treating high salinity wastewaters and RO brine.

However, MD has not been widely accepted in large-scale applications because there are still technical issues to be addressed. Problems that have been reported during the operation of the MD process include membrane fouling [3,4], back flux depending on the concentration of the inflow water [5,6], relatively high heat loss from modules [7,8], durability, and the hydrophobicity of the membrane to avoid the wetting phenomenon [9,10,11]. Moreover, MD flux should be further improved to gain economic competitiveness [12,13]. Several techniques have been developed to increase flux, including the optimization of operating parameters such as temperature and flow rate [12,13], development of novel configuration [13,14], and fabrication of innovative membrane materials [15,16,17].

When the operating conditions for MD are considered, previous works mostly focused on the effect of feed temperature, distillate (or cooling) temperature, and feed flow rate on MD flux. However, little attention was paid to the effect of external hydraulic pressure, which becomes an important issue during the scale-up of MD processes. Although MD processes should be designed to minimize the pressure difference across the membrane, it cannot be completely avoided in practical cases. Moreover, the pressure difference may be more problematic in DCMD configuration in which feed and distillate flows are in direct contact with the membrane. The difference in pressure across the membrane may result in membrane compaction and wetting. Nevertheless, little information is available on its effect on the performance of MD processes.

Accordingly, this study aims at systematic analysis and evaluation of the pressure effect on DCMD process. Bench-scale DCMD experiments were carried out to examine the effect of pressure on flux and rejection. The gas permeabilities of MD membranes were measured under various pressure conditions. A scanning electron microscope (SEM) was used to visually confirm the changes in membrane structures by the pressure. The possibility of local wetting caused by the pressure lower than liquid entry pressure was also examined.

## 2. Theory

### 2.1. Mass and Heat Transfer in Direct Contact Membrane Distillation (DCMD)

**Figure 1:**
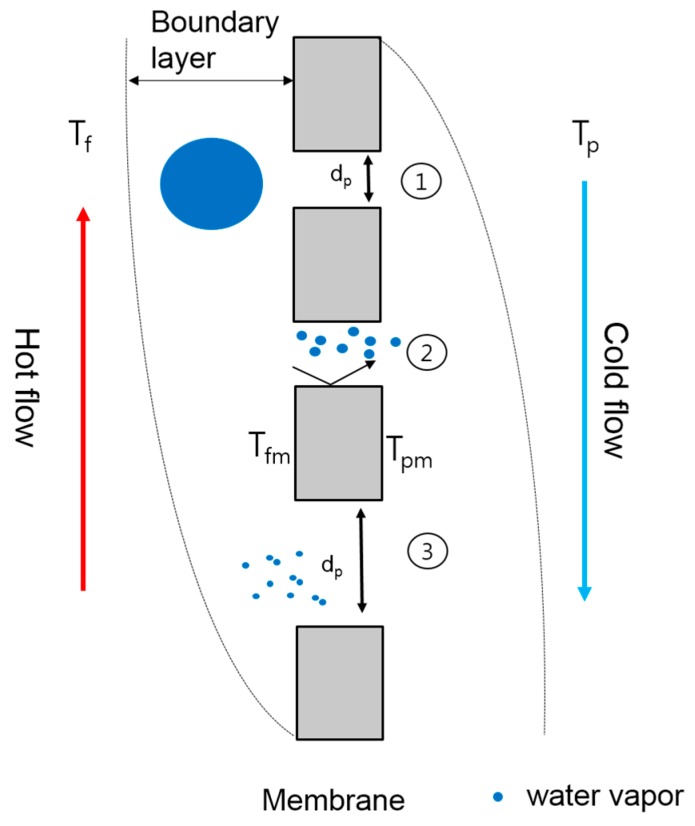
Basic principles of direct contact membrane distillation (DCMD) process.

Figure 1 illustrates the heat and mass transfer in DCMD. The temperature difference (*T_f_* − *T_p_*) exists across the membrane between the feed side (*T_f_*) and the permeate side (*T_p_*). At the membrane surface, water evaporates and transports through the membrane. Thus, two thermal boundary layers appear on both sides of the membrane. Within the boundaries, the feed temperature decreases from the bulk solution to the surface of the membrane (*T_fm_*). During the transfer of the latent heat from the feed when water vapor pressure condenses into the fresh water stream, the cold stream temperature (*T_p_*) increases and a boundary layer occurs from the surface of the membrane (*T_pm_*) to the bulk of the cold stream. Accordingly, the driving force of DCMD occurs by the temperature difference between *T_fm_* and *T_pm_*, which is less than the vapor pressure between *T_f_* and *T_p_*.

The mass transfer in MD is described by three models including molecular diffusion, Knudsen diffusion, and Poiseuille flow models. As listed in Table 1, different models should be applied depending on the Knudsen number (*Kn*), which represents the relation of the mean free path to the pore size of the membrane:

(1) $$Kn=\frac{\lambda }{d_{p}}$$

where *λ* and *d_p_* are mean free paths (transferred gas molecule) and mean pore diameter of the membrane, respectively. The mean free path of the molecule can be calculated using the equation:

(2) $$\lambda =\frac{K_{b}T}{\sqrt{2}\pi p\sigma^{2}}$$

where *σ*, *K*_b_, and *p* are the collision diameter of water vapor molecules, Boltzmann constant, and mean pressure in the membrane pores, respectively. For example, at the membrane temperature of 70 °C, the mean free path of water vapor is 0.1509 μm. If the mean pore diameters of membranes are 0.22 μm and 0.45 μm, respectively, the Knudsen numbers are 0.333 and 0.682, respectively. This implies that the Knudsen molecular diffusion transition model should be applied for those membranes.

The mass transfer by Knudsen diffusion is given by [16]:

(3) $$J_{K}=\frac{4}{3}d\frac{\varepsilon }{\delta \tau }\sqrt{\frac{M}{2\pi RT}}\Delta P_{A}$$

where Δ*P_A_* is the pressure gradient within the membrane pores, R is universal gas constant, *M* is molecular weight of the gas. *J_k_* is the vapor flux in the membrane resulting from Knudsen diffusion. δ, τ, and *T* are the thickness of membrane, the average tortuosity of the membrane and the average temperature in the pores, respectively. On the other hand, the mass transfer by molecular diffusion is given by:

(4) $$J_{M}=\frac{1}{1-y_{a}}\frac{\varepsilon D_{AB}}{\tau \delta RT}\Delta P_{A}$$

where *J_m_* is the vapor flux in the membrane resulting from molecular diffusion. *D_AB_* and *y_a_* are the diffusivity of water vapor and the mole fraction of the water vapor while Δ*P_r_* represents the partial pressure gradient of water vapor, which must be calculated based on the interfacial temperatures. The Antoine equation can be used to calculate the vapor pressures.

In the transition region, the total mass flux is correlated with *J_M_* and *J_K_* as follows [18]:

(5) $$\frac{1}{J_{M-K}}=\frac{1}{J_{M}}+\frac{1}{J_{K}}$$

Substituting Equations (3) and (4) into Equation (5) produces the following differential equation of mass flux for component *i* in the Knudsen molecular diffusion transition mechanism:

(6) $$J_{M-K}=\frac{\varepsilon }{RT\tau }{\left(\frac{3}{4d}\sqrt{\frac{2\pi M}{RT}}+\frac{P-P_{A}}{PD_{AB}}\right)}^{-1}\Delta P_{A}$$

where *P* is the total pressure within the pores.

### 2.2. Membrane Compaction

**Figure 2:**
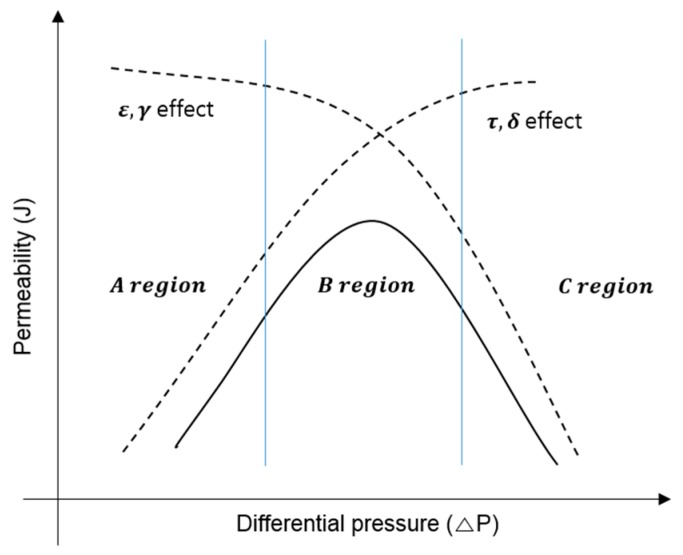
Compaction effect of membrane properties; the solid line is the experimental data. The dotted line is the theoretical value [20].

The parameters normally used to characterize a microporous membrane, such as porosity, average pore size, tortuosity, and thickness, influence the membrane permeability, and they are affected by membrane compaction. Figure 2 depicts how the competing effects of ε and r which are decreasing flux, and how τ and δ, which are increasing flux, affect overall permeability for the MD membrane [17,18,19]. The graph shows that the dominant compaction effect incorporates only one of the corresponding membrane parameters to suit experimental data, but the effects are not necessarily limited to that parameter.

Membrane compaction is generally described by measuring the changes in thickness (δ) in response to pressure (Δp). This can be expressed by the following equation [21]:

(7) $$\delta_{i}=\delta_{0}-\delta_{1}\Delta p$$

where $\delta_{i}$, $\delta_{0}$, $\delta_{1}$ are the initial membrane thickness, the uncompacted membrane thickness, and the compaction coefficient. Δp is the pressure drop across the membrane. The thickness of membranes can be directly measured using techniques such as a SEM.

In MD, gas permeation experiments are conducted to determine the characteristics of the porous membrane. The total mass flux is generally interpreted by the Knudsen diffusion–Poiseuille flow mechanism that is derived from Equations (3), (4) and (6) [21].

(8) $$J_{K-P}=\left(\frac{8}{3}\frac{\varepsilon r}{\tau \delta }\sqrt{\frac{1}{2\mu MRT}}+\frac{\varepsilon \gamma^{2}}{\tau \delta }\frac{1}{8\eta }\frac{P_{A}}{RT}\right)\left(P_{fm}-P_{pm}\right)$$

where *P_fm_* is the water vapor pressure at the membrane surface of the feed side, and *P_pm_* is that of the permeate side.

This can be rewritten as:

(9) $$J_{K-P}/\Delta p=A_{0}+B_{0}p_{m}$$

where *A*_0_ is the Knudsen flux coefficient, *B*_0_ is the viscous flux coefficient. In order to derive *A*_0_ and *B*_0_, a gas permeation experiment is carried out at various *P_m_*.

## 3. Materials and Methods

### 3.1. Membrane Characterization

Hydrophobic microporous flat sheet membranes, which are commercially available (Merck Millipore Ltd., Darmstadt, Germany), were used in this study. Four different membranes were selected, including HVHP, GVHP, FGLP, and FHLP. The first two membranes are made of polyvinylidene fluoride (PVDF) and their structures are asymmetric. The others are made of polytetrafluoroethylene (PTFE) and composed of an active layer and the support layer. The pore size, porosity, and thickness of each membrane are summarized in Table 1.

#### 3.1.1. Membrane Thickness and Pore Area

A field-emission scanning electron microscope (FE-SEM) (MIRA3 TESCAN) was used to measure the pore area and thickness of the membranes. The membrane samples were prepared with gold coating for 15 min. The SEM images were taken with magnifications ranging from 1000× to 20,000×. An operating voltage of 10 kV was used to accelerate the electron beam. SEM images were analyzed using Image J software (National Institutes of Health, Bethesda, MD, USA) to calculate pore size. Using automatic color threshold adjustment in Image J, the pores were automatically calculated.

#### 3.1.2. Contact Angle Measurement

Contact angle was measured to determine wetting properties of membrane surfaces. Liquid droplets were applied to the membrane surface using a syringe. The small droplet is observed using a digital camera. The image of the droplet is then analyzed by Image J (National Institutes of Health, Bethesda, MD, USA) from points marked along the droplet-air interface to calculate the contact angle at the droplet-surface interface.

### 3.2. Gas Permeability Measurements

**Figure 3:**
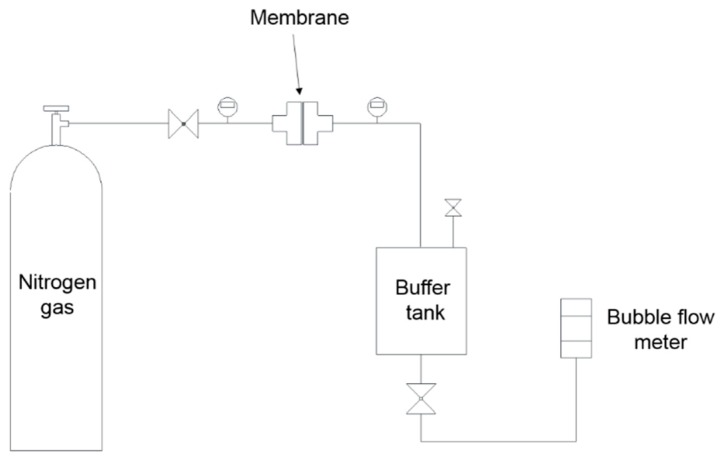
Schematic diagram of gas permeability measurement system.

The following procedures were proposed to examine the effect of hydraulic pressure on MD membrane properties. The results of gas permeability measurements were compared under low-pressure conditions (Δ*p* = 1 kPa) and high-pressure conditions (Δ*p* = 1 kPa). High-purity nitrogen gas was used for all the gas permeability experiments. Figure 3 shows the schematics of the experimental set-up.

#### 3.2.1. Gas Permeability Measurements at Low Pressure (Δ*p* = 1 kPa)

The method for gas permeability measurement was adopted by a previous study [20], which is briefly described as:

1. Each membrane was placed into the module of the gas permeability device.
2. Then, feed pressure (*P_m_*) was adjusted with the regulator of the membrane module and permeate pressure was allowed to come to a steady state and the buffer tank allowed the permeate-side pressure. This created a pressure difference between the membranes.

The gas flow rate was measured with an air flow calibrator. In order to reduce the deformation of the structure of the membrane, the pressure difference between both sides (Δ*p*) was adjusted by 1 kPa ± 0.5 kPa. The experiments used different membranes for each pressure test. The separating membranes used the average values among five samplings. The pressure was measured using an electronic manometer, which offer 0.5% full-scale accuracy (Dwyer, DPGAB-04, USA). The pressure at the feed side used stage two of the gas regulator (YUTAKA ENG., GSN2-4) and the permeate side adjusted fine pressure using micro-metering valves (HOKE, Milli-Mite 1300 Series). The transmitted flow rate was measured by the standard air flow calibrator (SENSIDYNE, Gilibrator-2). The system includes a highly-accurate (better than 1%), electronic flowmeter that provides instantaneous air flow readings and cumulative averaging of multiple samples. The results were measured 10 times and an average of the values was used. The graph was derived from the flow rate corresponding to the pressure obtained in the experiment.

Based on Equation (9), the intercept with y-axis was *A*_0_ and the slope was *B*_0_ and these were obtained by plotting the curve of *J_k-p_* with *P_m_*. The membrane properties such as *r* and *ε*/*τδ* were obtained from *A*_0_ and *B*_0_ by the following equations [1]:

(10) $$r=\frac{16}{3}\frac{B_{0}}{A_{0}}\sqrt{\frac{8RT}{\pi M}}\eta$$

(11) $$\frac{\varepsilon }{\tau \delta }=\frac{8\eta RTB_{0}}{r^{2}}$$

η is the gas viscosity.

#### 3.2.2. Gas Permeability Measurements at High Pressure (Δ*p* = 30 kPa)

Another sets of gas permeability experiments were carried out under high pressure conditions to examine the effect of compaction on the membrane properties [22,23]. Nitrogen is introduced from the high-pressure gas cylinder into the membrane module, in which the flat sheet membrane is placed on a porous metal to support the membrane. The pressure on both sides of the membrane are regulated by a gas regulator and measured with digital pressure sensors. For 5 min, the gas permeability of the membranes were measured with transmembrane pressure (Δ*p*) of 30 kPa. The experiments were repeated five times in each feed pressure (*P_m_*) to reduce experimental random errors.

### 3.3. DCMD Experiments

**Figure 4:**
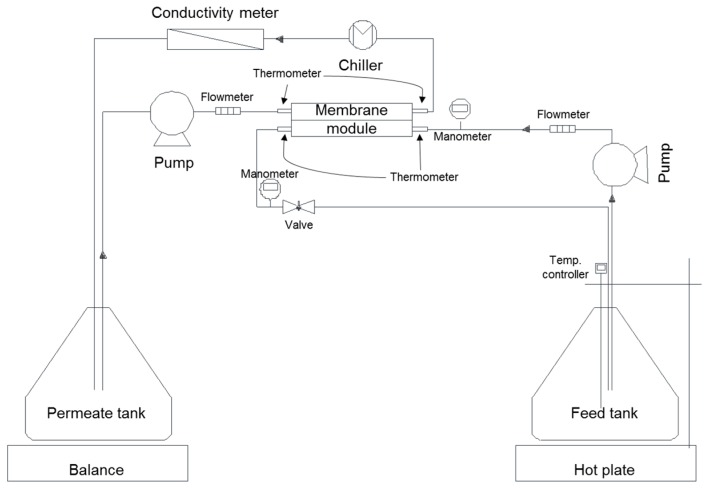
Schematic diagram of DCMD experiment set-up.

A plate-and-frame membrane module, which was especially designed to have channels on both sides of the membrane, was used for DCMD experiments. As illustrated in Figure 4, the cold water flowed on the distillate side and the hot solution flowed on the feed (active layer) side. The channel has dimensions of 60 mm in length, 15 mm in width, and 1 mm in height, providing an effective membrane area of 900 mm^2^. The volume of solution was 2 L which is on the feed and permeate side. The DCMD flat sheet module was made of acrylic to improve chemical stability.

Feed solution was continuously pumped from a feed tank through the tangentially oriented membrane module and back to the tank. The hot plate (IKA, C-MAG HS7, Staufen, Germany) was constantly heated to within ±0.5 °C of the desired feed temperature. The product water (distillate) was cooled by a water chiller (JEIO TECH, RW-0525G, Korea) within ±0.5 °C of the desired temperature. Feed solution inlet temperatures of 69.5 ± 0.5 °C and 70 ± 0.5 °C at velocities of 0.069 ± 0.015 m/s were also utilized. In all these experiments, the inlet temperature of the cold stream was controlled at 20 ± 2 °C. The brine feed was prepared by dissolving NaCl in deionized water to set the concentration of 3.5 g/L. The NaCl had a purity of 99% and was purchased from Sigma Aldrich, USA. The flux was determined by measuring the weight of the product tank and was calculated based on the membrane area. A conductivity meter in the product tank was used to monitor conductivity changes, which were used to monitor the wetting phenomenon.

To examine the pressure effect on DCMD flux, the feed pressure was adjusted using a micro-metering valve attached to the feed-side outlet. The pressure in the distillate side was maintained at the atmospheric pressure. The average feed pressure was adjusted from 5 kPa to 110 kPa. The maximum applied pressures were less than the liquid entry pressures (LEPs) for the membranes used in the experiments.

## 4. Results and Discussion

### 4.1. Scanning Electron Microscopy (SEM) Analysis

**Figure 5:**
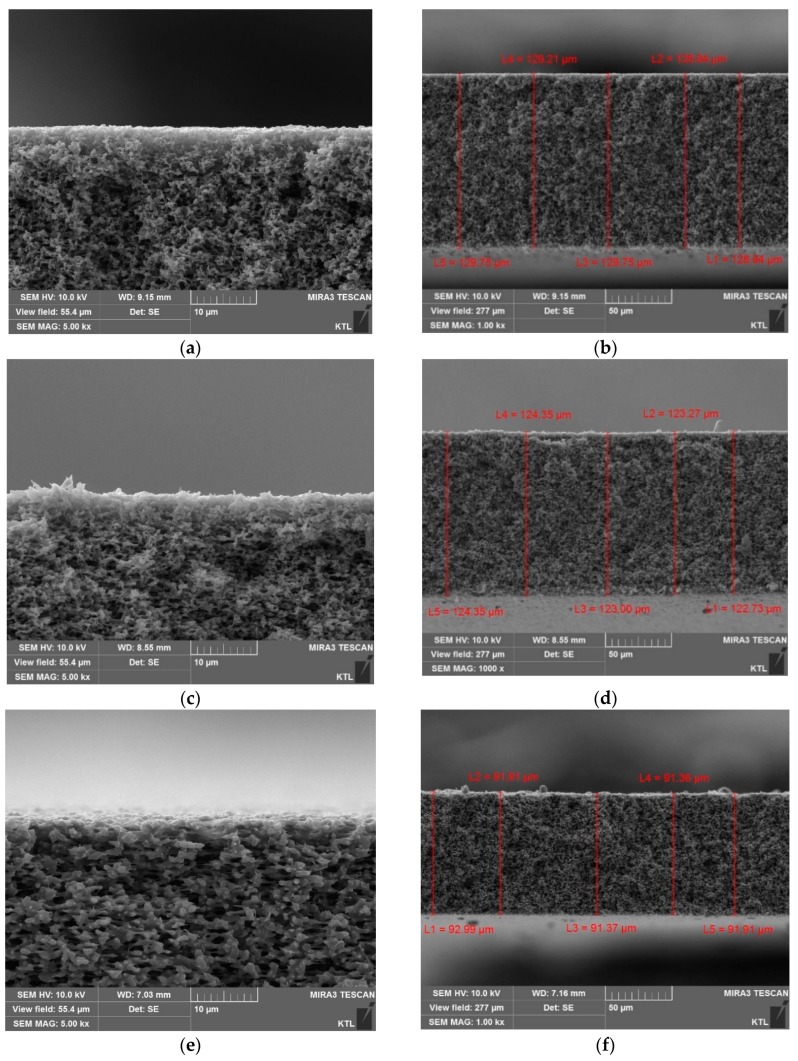

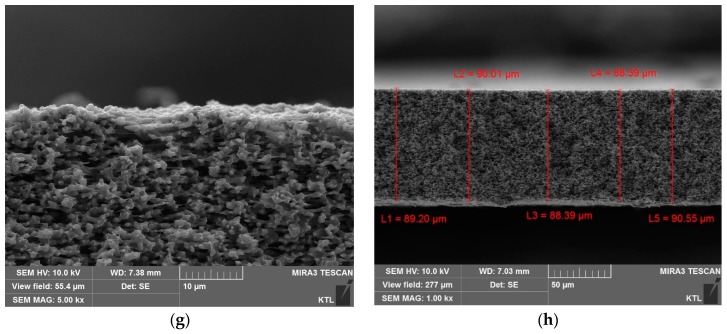
Scanning electron microscope (SEM) images for cross-sections of different polyvinylidene fluoride (PVDF) membranes. The labels (**a**), (**b**) are HVHP and (**e**), (**f**) are GVHP membranes without pressure, respectively. The labels (**c**), (**d**) are pressurized HVHP and (**g**), (**h**) are GVHP membranes at different pressure 30 kPa.

The cross sections and surfaces of the MD membranes were examined using SEM to analyze the effect of pressure on their structural parameters such as thickness and pore size. Figure 5a,b,e,f show the SEM images for the PVDF membrane cross-sections without the applied pressure. On the other hand, Figure 5c,d,g,h) depict the SEM images at the pressure of 30 kPa. The PTFE membranes were not used for this analysis because it was impossible to accurately measure the thickness of the active layer. The image analysis results indicate that the thicknesses of the membranes were reduced by 1.04% and 1.03%, respectively. These clearly suggest that the membrane compaction occurred under the pressure condition [24].

**Figure 6:**
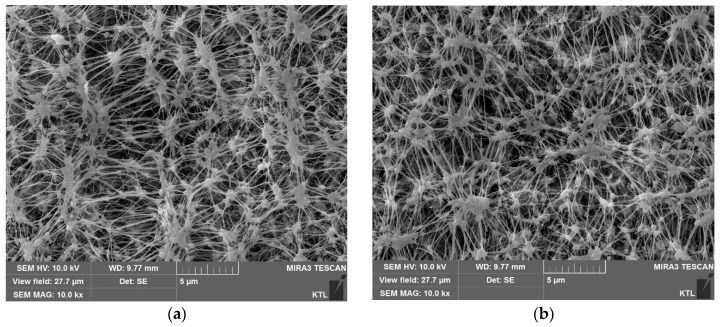

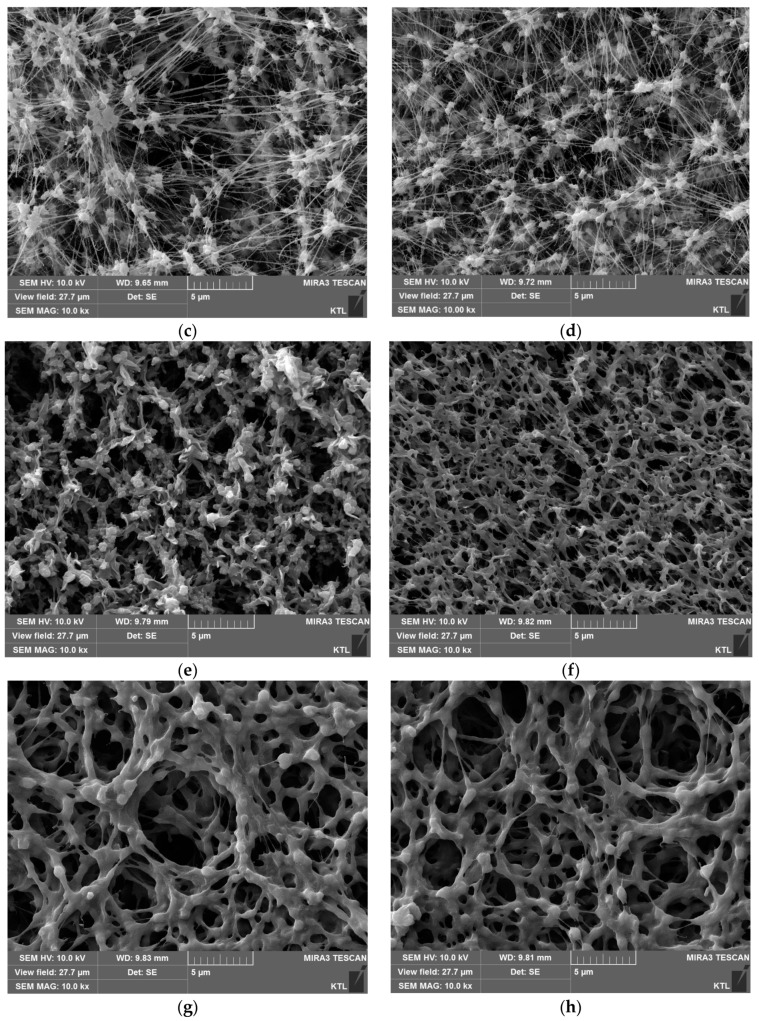
SEM images of polytetrafluoroethylene (PTFE) and PVDF membranes surfaces. The labels (**a**–**d**) are unpressurized FGLP, pressurized FGLP, unpressurized HVHP and pressurized HVHP, respectively. The labels (**e**–**h**) are unpressurized FGLP, pressurized FGLP, unpressurized FHLP and pressurized FHLP, respectively. The transmembrane pressure of membrane was set at 30 kPa.

The surfaces of the PVDF and PTFE membranes were also examined using SEM as illustrated in Figure 6. It is evident from these images that the size of surface pores was reduced by applying the pressure. For example, Figure 6a,b are the SEM images for the unpressurized and pressurized PVDF membranes (FGLP). As demonstrated, the pores were shrunk after applying the pressure. Similar results were observed for Figure 6c,d (HVHP), Figure 6e,f (FGLP), and Figure 6g,h (FHLP). These also imply that the membranes were deformed under the pressurized conditions.

Based on the image analysis of SEM images, the pore sizes of the membranes without the pressure were determined as shown in Table 2. The measured pore sizes of the membranes was similar to the nominal pore size provided by the manufacturer: 0.290 μm (measured, FGLP) vs. 0.20 μm (nominal, FGLP), 0.420 μm (measure, FHLP) vs. 0.50 μm (nominal, FHLP), 0.240 μm (measured, GVHP) vs. 0.22 μm (nominal, GVHP), and 0.480 μm (measured, HVHP) vs. 0.45 μm (nominal, HVHP), respectively.

### 4.2. Gas Permeability Measurements

#### 4.2.1. Gas Permeability Measurements at Low Pressure (Δ*p* = 1 kPa)

**Figure 7:**
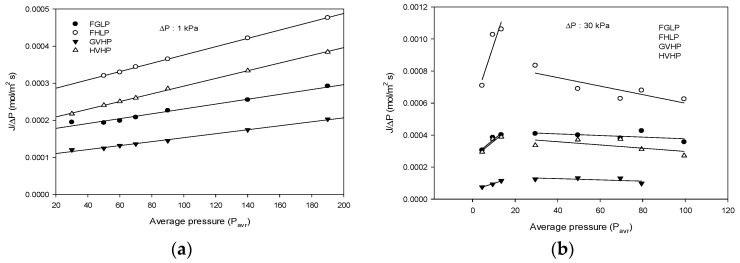
Dependence of *J*/Δ*p* on average pressure in gas permeability experiments. (**a**) Low Δ*p* (**b**) high Δ*p*.

The results of gas permeability measurements for membranes at various P_m_ values and the low transmembrane pressure (Δ*p* = 1 kPa) are shown in Figure 7a. As the pressure increases, the *J*/Δ*p* linearly increases. Using these results, the r and ε/τδ were determined using Equations (10) and (11) and are listed in Table 2.

#### 4.2.2. Gas Permeability Measurements at High Pressure (Δ*p* = 30 kPa)

Figure 7b shows the *J*/Δ*p* as a function of *P_m_* at the high transmembrane pressure (Δ*p* = 30 kPa). The dependences of *J*/Δ*p* were quite different between the low Δ*p* and the high Δ*p* conditions. Initially, the *J*/Δ*p* increases with an increase in the pressure (*P_m_*). Above the pressure of 30 kPa, however, the *J*/Δ*p* suddenly is dropped and then gradually reduced with the pressure. This indicates that the gas transport through the membrane is reduced by applying high *P_m_* (>30 kPa) and Δ*p* (30 kPa). Similar results in studies that were previously researched reflected a more physically rigid hollow fiber membrane that decreased the water permeability due to the increase in pressure [14].

The effect of high Δ*p* on gas permeability through the membranes can be explained by the deformation of the membrane structures. Using the results in Figure 7, *εγ*/*τδ* can be estimated based on Equations (10) and (11). When *P_m_* was below 15 kPa, the *εγ*/*τδ* of FGLP, FHLP, GVHP, and HVHP were about 2.51 $\times 10^{3}$, 4.7 $\times 10^{3}$, 0.24 $\times 10^{3}$, and 7.05$\times 10^{3}$, respectively. When P_m_ was between 30 kPa and 99.5 kPa, the *εγ*/*τδ* were about 3.13 $\times 10^{3}$, 7.61 $\times 10^{3}$, 1.57 $\times 10^{3}$, and 3.13 $\times 10^{3}$, respectively. It is evident from the results that the membrane structures were significantly modified by applying high Δ*p*.

### 4.3. DCMD Experiments

#### 4.3.1. Effect of Applied Pressure (Δ*p*) on Flux

**Figure 8:**
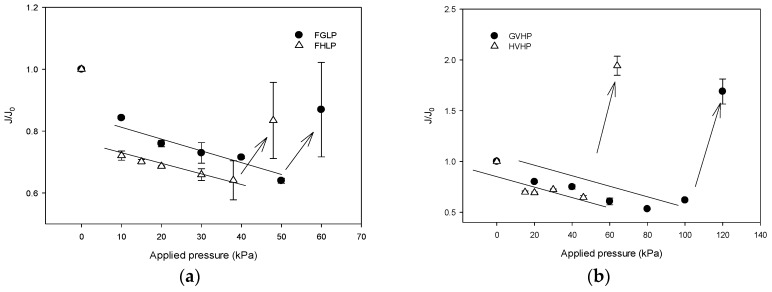
Dependence of *J*/*J*_0_ on applied pressure (Δ*p*) in DCMD system. (**a**) PTFE membranes (**b**) PVDF membranes.

A series of DCMD experiments were carried out using different MD membranes under a variety of applied pressure (Δ*p* conditions. Figure 8a shows relative flux (*J*/*J*_0_) as a function of Δ*p* for FGLP and FHLP membranes, which are made of PTFE. Initially, the *J*/*J*_0_ decreases with Δ*p*, suggesting that the membrane permeability is reduced by the applied pressure. The water permeabilities for FGLP and FHLP membranes were reduced by up to 15% and 25%, respectively. Above certain Δ*p* values (50 kPa for FGLP and 60 kPa for FHLP), however, the *J*/*J*_0_ suddenly increases, indicating the wetting of the membranes. It is unexpected that the wetting is observed below the LEPs, which are 208 kPa for FGLP and 124 kPa for FHLP. However, it is also likely that partial wetting of the membranes can occur due to their inhomogeneous properties [25].

Similar results were obtained for PVDF membranes (GVHP and HVHP) as demonstrated in Figure 8b. The water permeabilities for FGLP and FHLP membranes was reduced by up to 19% and 25%, respectively. When Δ*p* reaches critical values (120 kPa for GVHP and 60 kPa for HVHP), the *J*/*J*_0_ also suddenly increases. Again, it occurs below the LEPs (204 kPa for GVHP and 105 kPa for HVHP).

#### 4.3.2. Effect of Δ*p* on Salt Rejection

**Figure 9:**
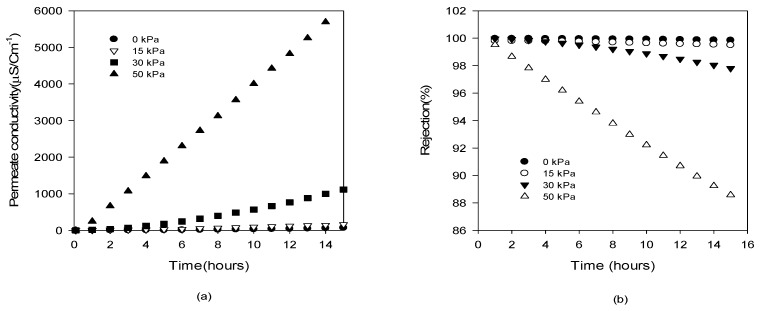
Dependence of distillate conductivity and solute rejection on time under various applied pressures (Δ*p*) (**a**) distillate conductivity (**b**) solute rejection.

In Figure 9, the distillate conductivity and NaCl rejection for the GVHP membrane are shown as a function of operating time at different Δ*p* conditions ranging from 0 kPa to 50 kPa. With an increase in Δp, the distillate conductivity increases more rapidly as shown in Figure 9a. Accordingly, the rejections were also affected by Δ*p*. As illustrated in Figure 9b, the rejection was almost 100% at Δ*p* = 0 kPa and 15 kPa. As Δ*p* increases, the rejection was reduced. For instance, after 15 h, the rejection was reduced to 89% at Δ*p* = 50 kPa. It should be noted that the LEP for this membrane is 204 kPa, which is approximately 4 times higher than this Δ*p*. Thus, it can be concluded that the membrane can be partially wetted under lower Δ*p* than the LEP.

### 4.4. Comparison of Mass Transfer Coefficients

The permeability of the MD membrane may be described by introducing the concept of the mass transfer coefficients, which is the ratio of flux (*J*) to driving force (vapor pressure difference). The mass transfer coefficient (*C_exp_*) for MD is defined by:

(12) $$C_{exp}=\frac{J}{P_{T_{f}}-P_{T_{p}}}$$

**Figure 10:**
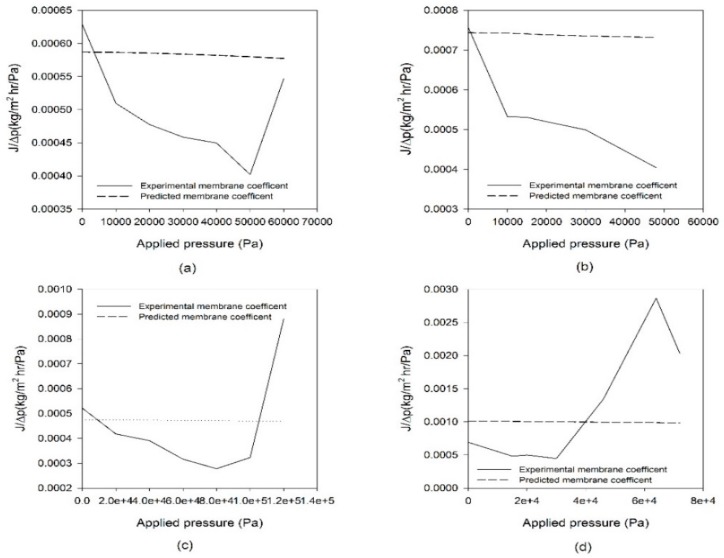
Comparison of mass transfer coefficients from model prediction and experimental measurement. (**a**) FGLP membrane, (**b**) FHLP membrane, (**c**) GVHP membrane and (**d**) HVHP membrane.

The mass transfer coefficient can be calculated either by theoretical model based on Equations (10) and (11) or from experimental data in Figure 8. The experimental and predicted mass transfer coefficients are compared in Figure 10. It seems that the mass transfer coefficients predicted by the model equations are quite different from experimental results. This suggests that the pressure effect cannot be easily explained by the conventional MD models. A novel model to consider the effect of Δ*p* on membrane structure and permeability will be required to predict these behaviors quantitatively.

## 5. Conclusions

In this study, the effect of transmembrane pressure (Δ*p*) on the properties and performance of MD membranes was investigated through gas permeability measurements and DCMD experiments. The following conclusions were made on the basis of the results of this study:

1. The results of SEM analysis indicate that the MD membranes are deformed by applying the hydraulic pressure across the membrane. The thickness, pore size, and porosity of the membranes were found to be reduced.
2. The gas permeabilities of the membranes were significantly reduced under high Δ*p* conditions. This is attributed to the deformation or compaction of the membrane structures.
3. A set of DCMD experiments was carried out by adjusting Δ*p*. The membrane permeability decreases with an increase in Δ*p*, which is attributed to the compaction of the membrane. When Δ*p* exceeds critical values, however, the membrane permeability abruptly increases, which results from partial wetting of the membranes.
4. Initially, the *J*/*J*_0_ decreases with Δ*p*, suggesting that the membrane permeability is reduced by the applied pressure. The water permeabilities for FGLP and FHLP membranes was reduced by up to 15% and 25%, respectively. Above certain ΔP values (50 kPa for FGLP and 60 kPa for FHLP), however, the *J*/*J*_0_ suddenly increases, indicating the wetting of the membranes. The partial wetting phenomena were also confirmed by monitoring the solute rejection by the membranes under various Δ*p*.
5. Under various Δ*p* conditions, the mass transfer coefficients determined from model equations were compared with those obtained from the experimental data. The model predictions failed to match the experimental results. This suggests that the current MD models cannot properly reflect the effect of Δ*p* on its performance.
6. Based on above results, it can be concluded that the control of feed pressure and transmembrane pressure are important not only in pressure-driven membrane processes but also thermal membrane processes such as DCMD. In fact, the MD pilot plant operated at about 30 to 70 kPa (Pilot Plant in Korea). Based on the results of this study, it is desirable to design the MD system to operate at 30 kPa or less.

## Figures and Tables

**Table 1:** Mass transfer models through the membrane.

Continuum Region	Transition Region	Knudsen Region
*Kn* < 0.01 or *d_p_* > 100*λ*	0.01 < *Kn* < 1 or *λ* < *d_p_* < 100*λ*	*Kn* > 1 or *d_p_* < *λ*

**Table 2:** Properties of hydrophobic membrane using DCMD tests.

Membrane Trade Name	Material	*δ* (μm)	*r_p_* (μm)	*ε* ^a^	$\frac{\varepsilon }{\tau \delta }$ (M^−1^)	*K*_1_ ($\frac{\varepsilon }{\tau }$)	LEP_w_ (kPa) ^a^
GVHP	PVDF	112	0.120 ^c^	0.75	2930 ± 20 ^b^	1520 ± 10 ^b^	204
HVHP	PVDF	130	0.240 ^c^	0.75	6130 ± 50 ^b^	2080 ± 20 ^b^	105
FGLP	PTFE/PE	130 ^a^	0.145 ^c^	0.70	7930 ± 50 ^b^	560 ± 6 ^b^	208
FHLP	PTFE/PE	175 ^a^	0.210 ^c^	0.85	10,880 ± 80 ^b^	1170 ± 10 ^b^	124

^a^ Reported in [2]. ^b^ Measured values by gas permeability (GP) test. ^c^ Measured values by SEM images.

## Nomenclature

*A*	Membrane Area
*A*_0_	Knudsen flux coefficient
*B*_0_	viscous flux coefficient
*D_AB_*	diffusivity of water vapor
*ε*	membrane porosity
*K_b_*	Botzmann constant
*σ*	collision diameter
*p*	Mean pressure
*J_K_*	Vapor flux in the membrane resulting from Knudsen diffusion
*R*	universal gas constant
*τ*	Average tortuosity
*T*	Average temperature in the membrane pores
*J_M_*	Vapor flux in the membrane resulting from molecular diffusion
*y_a_*	mole fraction of the water vapor
$\delta_{i}$	initial membrane thickness
$\delta_{0}$	uncompacted membrane thickness
$\delta_{1}$	compaction coefficient
*η*	gas viscosity
